# Health workers’ experience of providing second-trimester abortion care in Ethiopia: a qualitative study

**DOI:** 10.1186/s12978-023-01698-6

**Published:** 2023-10-17

**Authors:** Emily McLean, Astrid Blystad, Alemnesh H. Mirkuzie, Ingrid Miljeteig

**Affiliations:** 1 Bergen Center for Ethics and Priority Setting, Department of Global Public Health and Primary Care, University of Bergen, Årstadveien 21, 5020 Bergen, Norway; 2 Global Health Anthropology Research Group, Department of Global Public Health and Primary Care, University of Bergen, Årstadveien 21, 5020 Bergen, Norway; 3 John Snow Research and Training, Inc, Edna Mall Area, Addis Ababa, Ethiopia; 4 Institute for Health Metrics and Evaluation, University of Washington, 3980 15th Ave, Seattle, WA 98195 USA

**Keywords:** Second-trimester abortion, Abortion providers, Ethical dilemmas, Ethiopia, Qualitative, Sexual and reproductive health

## Abstract

**Background:**

Second-trimester abortions are less common than abortions in the first trimester, yet they disproportionately account for a higher burden of abortion-related mortality and morbidity worldwide. Health workers play a crucial role in granting or denying access to these services, yet little is known about their experiences. Ethiopia has been successful in reducing mortality due to unsafe abortion over the past decade, but access to second trimester abortion remains a challenge. The aim of this study is to better understand this issue by exploring the experiences of second-trimester abortion providers working in Addis Ababa, Ethiopia.

**Methods:**

A qualitative study with 13 in-depth semi-structured interviews with 16 health workers directly involved in providing second-trimester abortions, this included obstetrician and gynaecologist specialists and residents, general practitioners, nurses, and midwives. Data was collected at four public hospitals and one non-governmental clinic in Addis Ababa, Ethiopia and analysed using Malterud’s text-condensation method.

**Results:**

The providers recognized the critical need for second-trimester abortion services and were motivated by their empathy towards women who often sought care late due to marginalisation and poverty making it difficult to access abortion before the second trimester. However, service provision was challenging according to the providers, and barriers like lack of access to essential drugs and equipment, few providers willing to conduct abortions late in pregnancy and unclear guidelines were commonly experienced. This led to highly demanding working conditions. The providers experienced ethical dilemmas pertaining to the possible viability of the fetus and women desperately requesting the service after the legal limit.

**Conclusions:**

Second-trimester abortion providers faced severe barriers and ethical dilemmas pushing their moral threshold and medical risk-taking in efforts to deliver second-trimester abortions to vulnerable women in need of the service. Effort is needed to minimize health system barriers and improve guidelines and support for second-trimester abortion providers in order to increase access and quality of second-trimester abortion services in Ethiopia. The barriers forcing women into second trimester abortions also need to be addressed.

## Background

Second-trimester abortions are less common than abortions in the first trimester, yet they disproportionately account for a higher burden of abortion-related mortality and morbidity worldwide [1]. Young and poor women are especially vulnerable to suffering these harsh consequences [2, 3].

There are many and complex reasons explaining why women have second-trimester abortions; financial barriers, difficulty in finding services, uncertainty about the decision to abort and fetal anomalies have been documented [3–6]. The World Health Organization (WHO) has recently recognized the need for abortion services throughout pregnancy. Their new and updated guideline calls for the full decriminalization of abortion [7].

However, access to abortion after the first trimester of pregnancy remains limited in many countries [8–10]. As the pregnancy progresses and the fetus becomes larger, more are hesitant to accept abortion [11, 12]. Ethical disagreements over the potential viability and moral status of the fetus become central arguments for limiting abortions later in pregnancy [11, 12].

African countries are particularly confronted with high mortality from unsafe abortions and second-trimester abortions are likely to make up a disproportionate percentage of these [1, 13, 14]. Ethiopia stands out in this context with its political commitment to improve access to safe abortion care. Ethiopia revised its abortion law in 2005 in order to tackle the high number of women dying due to unsafe abortion [15].

**Fig. 1:**
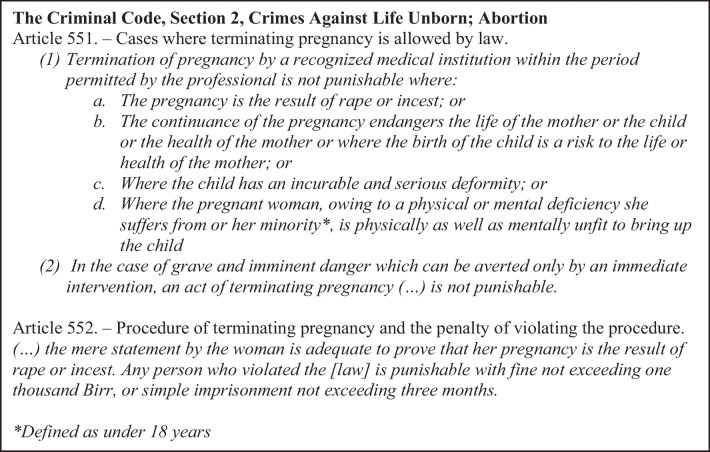
The Ethiopian abortion law

Abortion remains illegal in Ethiopia, but several exceptions have been made to this provision as seen in Fig. 1. The decade after the law was changed has demonstrated a drastic decline in mortality due to unsafe abortion [16, 17].

New abortion guidelines were issued in conjunction with the law, and specific training manuals for second-trimester abortion were released in 2021 [15, 18, 19]. The upper limit for abortion in Ethiopia is today 28 weeks of gestation, with second-trimester abortion defined as an abortion between 12 and 28 weeks of gestation [15].

Nonetheless, women are still suffering from unsafe abortion in Ethiopia and access to second-trimester abortions has been reported to be low and declining [16, 20]. Providers’ negative attitudes towards women seeking abortion care and refusal to serve them create severe demand for providers and challenges with the accessibility and safety of the service [21].

Providing abortion services in an Ethiopian context is demanding as providers have to balance abortion-related stigma, legal limits, conflicting personal values, and the knowledge that women may die due to unsafe abortion if denied the service [22]. With many women receiving information about abortion from their health care provider, the importance of providers in both granting and denying access to abortion services seems critical [23].

There is little knowledge about how second-trimester abortion services are delivered on the ground in Ethiopia. By describing providers’ experiences with delivering second-trimester abortion, the objective of this study is to gain a better understanding of the service provision and the experienced dilemmas. We hope this can bring valuable insights to improve training and guidelines.

## Methods

### Research design and aim

An explorative qualitative study interviewing second-trimester abortion providers with the aim to understand their experiences, dilemmas, and challenges in delivering the services.

### Study setting

Data was collected in Addis Ababa, the capital city of Ethiopia in 2022. Even though the majority of the 117,000 million large population lives in the rural parts of the country, the majority of abortions take place in urban centres [17, 24]. Addis Ababa has the highest abortion rate in the country estimated at 92 per 1000 women of reproductive age, compared to the country’s average of 24 per 1000 [17, 25]. This discrepancy has partly been explained by the fact that many women travel to the cities for abortion due to better service availability [17].

Second trimester abortions are to be provided at secondary- and tertiary-level hospitals and non-governmental organization (NGO) clinics with the appropriate set-up [15]. The procedure is either medical or surgical. Medical abortion can be conducted up to 28 weeks of gestation with the use of the medication Mifepristone and Misoprostol [19]. Surgical abortion is conducted with dilatation and curettage (D&C) and can only be used between 13 to 18 weeks of gestation [18].

Emergency surgical officers, general practitioners and obstetricians and gynaecologists are permitted to provide medical second-trimester abortion, whilst surgical second-trimester abortions can only be provided by specifically trained obstetricians and gynaecologists [15]. Midwives, nurses and health officers may assist in the surgical procedure [15].

### Sampling and recruitment

Healthcare workers directly involved in providing second-trimester abortion services at the time of the data collection were included in the study. These were seven obstetrician and gynaecologist specialists, four obstetrician and gynaecologist residents, three general practitioners, one nurse, and one midwife working at four different public hospitals and one NGO clinic in Addis Ababa. Health workers not involved in second trimester abortion provision either due to lack of training or not wanting to provide the service were not included.

The first study participants were identified in collaboration with our partners at Addis Centre for Ethics and Priority Setting and approached by the first author of this paper. Through these first contacts, snowballing was used to further identify participants. A text message or email was sent out to potential participants informing about the study and asking for participation. All potential participants approached except one agreed to participate in the study.

### Data collection

Data were collected by the principal investigator between March and April 2022. In total 13 in-depth semi-structured interviews, lasting 30–50 min each, were conducted with 16 providers at five different health facilities. The interview guide focused on the experience of providing second-trimester abortion, including potential dilemmas and challenges. Most interviews were conducted in English by the main investigator. One interview was conducted in Amharic with a study assistant and translator and the main investigator present. All interviews except three were individual interviews. The exceptions were conducted with two participants together at their request. The interviews were recorded and transcribed verbatim.

### Data analysis

Data were analysed using Malterud’s systematic text-condensation which draws upon grounded theory [26]. The preliminary analysis started during fieldwork through the recognition of emerging themes, and included adjustments to the interview guide. After all the data was collected the material was analysed through Malterud’s four steps. The first step involved reading the full material, then preliminary overarching themes were detected and discussed with the co-authors. In the second step meaning units were identified and coded. The third step involved sorting and condensing the codes into groups of common themes. In this step the themes that had first emerged were adjusted and rephrased. In the fourth and final step a recontextualization of the codes was done resulting in the write up of the final themes. These analysis steps were carried out by the principal investigator in collaboration with the research team to gain inter-coder reliability. Nvivo12 software was used to organize the material [27].

### Ethical approval

The study was approved by the Regional Ethical Committee in Norway (ID-no: 249828) and the Ethiopian Public Health Institute Institutional Review Board (ID-no: EPHI-IRB-402-2021). Written informed consent was obtained from the study participants. A small monetary gift was provided as compensation for the time spent doing the interview. Due to the sensitivity of the topic and the small number of providers involved in second-trimester abortion in Addis Ababa, a detailed description of the participants is not be provided to protect the anonymity of the study participants.

## Results

In our material, three main themes emerged. The first theme demonstrates the providers’ understanding of women’s need for second-trimester abortions. The second theme illustrates the many barriers to service provision that the providers had to deal with, like lack of access to essential equipment and drugs. The third theme points to ethical dilemmas the providers had to navigate, dealing with the emotional difficulty of conducting late abortion procedures and engaging with women who were presenting late and at times too late for abortion services.

### The need for second-trimester abortion services

The need for second-trimester abortion services was recognized by all the providers. Women coming for the service were described as vulnerable, commonly being “young”, “poor”, “students” or “living on the street”. The providers were acutely aware of the fact that they were among the few willing to conduct second-trimester abortions, hence making their decision vital for the women.

Lack of access to contraception, first-trimester abortion, and inadequate knowledge about pregnancy symptoms and abortion services, were among the reasons why women were not accessing abortion care earlier.

*«They don´t know the legal reasons for safe abortion, or that they can get safe abortion freely. They think they need to have some amount of money” (#4, OBGYN)*

*«They think it will take days and they cannot spend the night at the hospital without telling their parents” (#3, OBGYN)*

Several providers explained that the shame of pregnancy outside of marriage led women to deny the fact that they were pregnant in fear of stigma and social exclusion. These serious concerns again led to indecisiveness and delays in their abortion related decision.

*«In Ethiopia getting pregnant before marriage is another story, it causes you to be isolated from your community (…) it will be shameful, so they wouldn´t dare to go here and there to ask for abortion” (#7, OBGYN resident)*

*“Most of our women, our sisters in the society, they cover most of their problems, and when their abdomen is big and when the society see… They come late because of this» (#10, OBGYN resident)*

Fetal anomalies, like anencephaly and spinal bifida, were also noted as a reason for second-trimester abortion that most providers had encountered.

*“Some congenital abnormalities are diagnosed late. That results in a late referral from the health centre, and so they will be late in their decision about termination" (#10, OBGYN resident)*

The existential dilemmas confronting women with an unwanted pregnancy, which often led to severe delays in seeking help, were recognized by the providers and created a powerful motivation and an experience of offering lifesaving services.

*«We feel happy because if we do not help her, she might die. If we would not help her, she will go somewhere else, practice the traditional abortion by a different method and endanger herself» (#6, Midwife)*

### Barriers to service provision

Various barriers made providing second-trimester abortion difficult. Many providers described how the lack of health workers trained in second-trimester abortion, particularly in the surgical procedure, and the lack of hospitals with an adequate set-up for service delivery, were major barriers.

*“There are some hospitals that can provide the service and support clients, but there are very few. Outside of Addis it is still a challenge» (#2, OBGYN)*

*«There is the limitation of providers trained in surgical abortion, I think in Addis there are only three or four providers that can do D&C [surgical abortion]. That is not enough, and it is only available in one or two hospitals” (#8, OBGYN resident)*

*«We see clients who are referred to us from a two-day drive away» (#3, OBGYN)*

Another barrier that providers had experienced was the frequent stock-out of medicines and equipment. This meant that patients themselves had to purchase such items which few could afford.

*«Sometimes we even lack gloves, the antibiotic (…) the instruments, especially the surgical abortion ones, in some places they don´t have any instruments» (#12.2, OBGYN)*

*«Especially cervical drugs like laminaria [a drug used to prepare the cervix before a surgical abortion] are missing” (#13, OBGYN)*

*«When there is stock out from the government, the private pharmacies increase their price unfairly. Sometimes we might raise money for them (women seeking abortion), it is something you feel good about so that they can continue their education, and get back to their normal life» (#4, OBGYN)*

Unclear guidelines for the dosage of medical abortion and the lack of training in surgical procedures were other experienced barriers. This led to fear of complications and an experience of insecurity in their work.

*«Technically they are not as simple as first trimester abortions. Every week everything increases, and you also know that the complications increase every week” (#11, OBGYN)*

*“Misoprostol is a dangerous drug, which can end up with a uterine rupture, so sometimes if the gestational age is 26 weeks and the fundal height is around 27, the dose is challenging, there is no well-stated dose» (#9, OBGYN resident)*

These barriers often prevented the providers from giving optimal care and led to an experience of medical risk-taking.

### Ethical concerns and dilemmas

Most providers acknowledged the ethical challenges of abortion in general but highlighted that providing second-trimester abortion raised new and challenging ethical dilemmas. The desperation of women coming late for the service and their knowledge that women could end up having unsafe abortions if refused the service were difficult to be confronted with. This was especially true in encounters with women coming after the legal limit of 28 weeks of gestation.

“*When you start to tell her that she's beyond what's acceptable, she walks away leaving her phone and all her belongings behind. They just walk away and say, «I am going to commit suicide» (#12.1, OBGYN)*

*“They say that we are wrong about the estimated gestational age. Then we will tell them it is dangerous to go to another place to terminate this pregnancy» (#3, OBGYN)*

Doing the actual abortion procedure was in itself described as emotionally demanding, especially the more hands-on surgical abortion procedure in higher gestational week pregnancies. Some providers questioned if they were doing the right thing.

*“It’s very difficult by the way. Especially when you do the surgical one. The medical [abortion] we are not watching» (#12.2, OBGYN)*

*“It's a kind of roller coaster, sometimes you feel that you are doing the right thing helping these women, but sometimes you feel like "what am I doing?" (#3, OBGYN)*

How to handle the fetus after the abortion and dealing with questions of the possible viability of the fetus was particularly hard to be confronted with. Many found this the hardest part of their job.

*“If someone has been raped, we do not expect them to carry that baby until birth, right, it is psychologically traumatizing. But when you see the fetus, they look like human beings, you do not want to see that, right?” (#5.1, General practitioner)*

*«Whenever you see a large fetus expelled, your first like reaction is resuscitation (…) You don’t know what to do” (#3, OBGYN)*

The providers pushed their moral threshold to assist women in their desperate quests for second-trimester abortions, as they feared other solutions could seriously endanger these women’s lives.

## Discussion

Our study found that second-trimester abortion providers in Addis Ababa recognized the importance of their work and understood the critical need for the services they provided. Yet, several barriers like lack of access to essential drugs and equipment, scarcity of providers willing to conduct second-trimester abortions and unclear guidelines made service provision difficult. This led to highly demanding working conditions and an experience of insecurity in their work. They faced challenging ethical dilemmas having to deal with women coming with high gestational age pregnancies, and the emotional and ethical difficulties of conducting the abortion procedure itself.

To our knowledge, our study is the first that looks in-depth at the provision of second-trimester abortion from the perspective of the providers in Ethiopia. In line with other studies, the providers in our study explained how women often end up having abortions in the second trimester due to the social stigma associated with pregnancy outside of marriage, lack of ready access to contraception and first-trimester abortion, and implications of poverty and inadequate knowledge about pregnancy symptoms and abortion services [3–6, 28]. There is an urgent need to address such structural barriers to reduce the number of women who do not receive adequate abortion care.

We found that the providers had substantial empathy with and understanding for women seeking second-trimester abortion services. Many thus pushed their ethical threshold and medical risk-taking to help these women. Such scenarios have similarly been seen in studies from Senegal and Argentina, where health workers when confronted with legal restrictions on abortion tried to assist women getting access to care or prevent them from prosecution [29, 30]. These examples demonstrate the critical role health workers play in granting women access to abortion services, though also the potential power they have in refusing the service.

Several interconnected health system barriers linked to a lack of resources and a scarcity of providers willing to offer second-trimester abortion services were found in our study. Especially the severe lack of providers trained or willing to conduct surgical abortions were implied challenges to service provision. Evidence from South Africa shows that recruiting abortion providers is generally difficult and becomes particularly demanding for the provision of second-trimester abortion [11, 21, 31]. Our previous study from Ethiopia found that abortion providers often faced stigma from colleagues and the community, forcing them to hide the content of their work from friends and family [22]. While the powerful stigma associated with the service is likely to be a contributing factor, more research is needed to better understand the mechanisms behind why so few health workers are willing to offer second-trimester abortion services.

Carrying out the abortion procedure, and not the least being confronted with women arriving at the health facility after the legal limit had passed was experienced as emotionally and ethically difficult for the providers in our study. Ethical doubts tied to the size of the fetus as the abortion approaches the viability threshold has also in other settings been found to make providers uncomfortable, unsure and making it more likely that they decline to provide the service [21, 32]. The hands-on procedure of surgical abortion was pointed out by our study participants to be particularly emotionally difficult and medical abortion was seen by some doctors as easier as they were less involved because the nurses were following the women more closely. This has also been found in other studies [33, 34]. This is concerning as there is a need for surgical abortion procedures in certain situations, even though medical abortion is becoming increasingly common.

The issue of how to handle the aborted fetus was raised by several of the providers in our study and is a topic that is rarely talked about in public as well as in academic settings [12, 33]. There is a fear that talking too openly about the more visceral parts of abortion care may lead to a potential backlash against abortion services [12]. This does however leave the providers without guidance and support in the handling of the abortion care.

The Ethiopian training module for medical second-trimester abortion mentions the possibility of experiencing transient signs of fetal life after the abortion and recommends the induction of fetal asystole [19]. The new WHO guidelines also provide a regiment, but access to the drugs and the training needed for this procedure is not commonly available. The providers in our study describe how handling the fetus was the hardest part of their job and that they were unsure how to deal with it. It seemed that they were often left to deal with such dilemmas on their own, and it is reasonable to believe that many were reluctant to engage in second-trimester abortion because of this particular part of the challenge. Better support and clear guidelines on how to handle emotions and dilemmas that come with providing second-trimester abortion are thus needed to better secure safety and access.

### Strengths and limitations

There is a lack of studies exploring second-trimester abortion service delivery from the providers’ perspective, especially from under-resourced healthcare systems like in Ethiopia. With the central role health workers play in granting and denying women access to safe abortion services, gaining a better understanding of their experiences seems crucial for further development of training programs and policy.

The focus on Ethiopia is illuminating due to the country’s revised abortion law and the estimated decrease in deaths due to unsafe abortions over the past decade. This has led many countries to look to Ethiopia and to learn from their experience and find guidance on the legal revision and provision of abortion care services. With the continues burden of unsafe abortions in low-income contexts, more knowledge is needed on how to enhance the conditions for the health care providers to reduce the enormous strain placed upon them.

The study was limited to second trimester abortion providers in Addis Ababa due to the COVID-19 pandemic and the ongoing conflict in Ethiopia at the time of data collection. Although it may be assumed that many of the challenges abortion providers experience in other parts of the country are similar to the findings presented in our study, distinct issues may emerge in the rural parts of the country due both to the increased visibility and to the reduced accessibility to safe abortion services outside the urban centres.

The majority of the participants in our study were obstetricians and gynaecologists and physicians. Nurses and midwives are also involved in second-trimester abortion care provision and are often the ones following the women through a medical abortion. Future studies should look deeper into their perspectives, as well as that of other staff at the hospital.

The principal investigator is from Norway, a context in which abortion is readily available and less stigmatized. Although Ethiopian nationals are part of the study team, the positioning of the principal investigator vis a vis the study participants need to be considered in assessment of the findings. Abortion has remained a highly sensitive topic, and the study findings must be assessed in this light.

## Conclusion

Despite Ethiopia’s substantial efforts to improve access to abortion services, with the aim to reduce the high maternal mortality ratio in the country, there is still considerable demand for safe and available second-trimester abortion services. Hence, the present study findings suggest that health systems barriers must be minimalized, and the providers’ ethical concerns need to be taken seriously through improvements in guidelines and support. The many unequitable barriers pushing women into second-trimester abortion need to be urgently addressed, primarily by increasing the attention to universal access to contraception and first-trimester abortion.

## Data Availability

The data analyzed during the current study are not publicly available due to the sensitivity of the research subject and the ethical importance of protecting the anonymity of the study participants.

## Abbreviations

WHO: World Health Organization
NGO: Non-governmental organization
D&C: Dilatation and curettage
